# Effects of TLR7 Polymorphisms on the Susceptibility and Progression of HIV-1 Infection in Chinese MSM Population

**DOI:** 10.3389/fimmu.2020.589010

**Published:** 2020-10-26

**Authors:** Tong Zhang, Junping Zhu, Bin Su, Lina Cao, Zhen Li, Huanhuan Wei, Xiaojie Huang, Kai Zheng, Aixin Li, Ning Chen, Lifeng Liu, Wei Xia, Hao Wu, Qiushui He

**Affiliations:** ^1^ Center for Infectious Diseases, Beijing Youan Hospital, Capital Medical University, Beijing, China; ^2^ Department of Medical Microbiology and Research Centre of Microbiome, Capital Medical University, Beijing, China; ^3^ Department of Medical Microbiology and Immunology, University of Turku, Turku, Finland

**Keywords:** toll-like receptor 7, polymorphism, HIV-1, progression, viral load, set point

## Abstract

Toll-like receptor (TLR) 7 plays a key role in innate and adaptive immunity for HIV-1 infection. We evaluated the effect of TLR7 polymorphisms on disease susceptibility and progression of HIV-1 infection in Chinese MSM (men who have sex with men). Blood samples were taken from 270 patients with laboratory confirmed HIV infection, 196 male controls were tested, and three TLR7 intronic polymorphisms (rs179010-C > T, X:12884766; rs2074109-T > C, X:12885330; and rs179009-A > G, X:12885361) were analyzed by PCR-based sequencing. The frequency of TLR7 rs179010 T allele was significantly lower in MSM patients than in controls (P = 0.039). The haplotype TTA was associated with a decreased susceptibility to HIV-1 infection (P = 0.013), especially to acute HIV-1 infection (AHI) (P = 0.002), but not to chronic HIV-1 infection (CHI). Furthermore, the haplotype TTA is linked to slow disease progression in AHI patients (P = 0.002) and a lower viral load (P = 0.042). In contrast, TLR7 rs179009 allele A contributed to a higher set point in AHI patients with rapid progression, and the frequency of rs179009 minor allele G was over-presented in CHI patients. This finding supports a role for genetic variations of TLR7 in susceptibility and disease progression of an HIV-1 infection in Chinese Han population and warrants further studies on the effect of TLR7 polymorphisms on HIV-1 infection in different populations.

## Introduction

Although the global human immunodeficiency virus (HIV) epidemic is easing, men who have sex with men (MSM) remain at high risk of infection (1). Data from China show that the proportion of reported HIV cases infected through sexual contact has increased from 33% in 2006 to 76% by 2011, of which 14% cases were attributable to MSM (2, 3). The latest report showed that the overall national prevalence of HIV among MSM from 2001 to 2018 was estimated to be 5.7% and the study showed an increased tendency in the HIV prevalence as time progressed (4). China’s HIV burden among MSM appears to present an increasing national public health challenge. Huang reported that a significantly faster CD4^+^ T-cell decline was found in a Beijing MSM cohort (5). And Chen found a dynamic change of monocyte subsets and their surface markers in acute and chronic HIV-1-infected MSM individuals, indicating that dynamic states of immune response occurred during the different clinical stages (6).

Interaction between the virus and the host is central in determining the outcome of HIV infection. TLRs stimulate the innate immune response by recognizing conserved motifs of invading microorganisms and promote subsequent adaptive immune responses (7). A number of studies have provided evidence that most TLRs including TLR7 played a functional role in an HIV infection (8–14). TLR7 can recognize RNA of various viruses including HIV (15–17). TLR7 is mainly expressed on intracellular compartments of plasmacytoid dendritic cells (pDCs) and is involved in the regulation of innate immune response *via* MyD88-dependent proinflammatory signaling cascades (18).

Growing data suggest that single nucleotide polymorphisms (SNPs) of TLRs are linked to susceptibility and progression of different infectious diseases. Several polymorphisms in different TLRs have shown their effects on HIV acquisition or disease progression (19–24). The study of a functional TLR7 variant (rs179008) in 2009 reported that TLR7 Gln11Leu was associated with susceptibility to and a more severe clinical course of HIV-1 disease (25). Valverde-Villegas has reported that the polymorphisms of TLR7, TLR8, TLR9 contributed to susceptibility to HIV infection in Brazilian individuals with European and African descendants, which highlighted the influence of ethnic background on the susceptibility to HIV infection (26). So far, there has been no report on the association of TLR7 SNPs and HIV infection in Chinese people. On the other hand, associations between TLR7 polymorphisms and HCV infection have been reported in Chinese people (27, 28). Our team had reported TLR7 intronic polymorphisms (rs179009 and rs179010) play an important role in susceptibility to and disease progression of a chronic hepatitis B virus (HBV) infection in male Chinese adults (29). We hypothesized these variations of TLR7 might influence the susceptibility to and progression of an HIV-1 infection. Here we show the associations between these intronic variations of TLR7 and the HIV-1 infection as well as the individual disease progression by adjusting viral load or set point in different clinical courses.

## Materials and Methods

### Study Subjects

A total of 466 study subjects including 270 MSM patients with confirmed HIV-1 infection recruited from March, 2010 to October, 2013 in Beijing Youan Hospital, and also 196 male controls who attended the annual healthy examination in 2015. Sixty-seven HIV-1-infected MSM patients accepted antiretroviral therapy (ART) immediately after enrollment. There are 157 MSM patients with AHI (including 114 ART naïve patients) and 113 MSM patients with CHI (including 89 ART naïve patients). The AHI patients were recruited from an HIV-1-negative high-risk MSM cohort that was screened every 3 months to detect seroconversion for HIV-1 infection at Beijing Youan Hospital (30). The AHI patients of naïve ART were further divided into groups of rapid progression and slow progression according to the criteria that either CD4<350 cells/μl occurred during the 2-3 following years of natural history, or baseline CD4 was below 350 cells/μl, which was defined as fast progression. In total there are 54 AHI patients classified with rapid progression and 60 AHI ones with slow progression. Another group, one hundred thirteen chronic HIV-1-infected patients were randomly enrolled from the MSM cohort of Beijing Youan Hospital. All HIV-1-infected patients in the study were MSM without HBV/HCV co-infection and other comorbidities. None of them were drug users. Chronic HIV-1-infected patients were diagnosed at least 1.2 years (median 1.8 years) before enrollment.

Some clinical parameters were detected for the AHI patients, including initial CD4 T-cell counts (ICD4), viral load, set point and the ratio of CD4 versus CD8 T cell count (CD4/CD8). The median and age range of patients with AHI (N = 157), CHI (N = 113) and controls (N = 196) was 32 years (20-53 years), 31 years (21-60 years) and 40 years (21-60 years), respectively.

### Ethics Statement

All the participants were provided written informed consent for participation in the study and for the storage and use of their clinical samples for research. This study and other related experiments were approved by the Beijing Youan Hospital Research Ethics Committee, and written informed consent was obtained in accordance with the Declaration of Helsinki. The study was carried out in accordance with approved guidelines and regulations.

### TLR7 Genotyping

Genomic DNA was extracted from blood using a commercial kit according to the manufacturer’s instructions (QIAGEN DNA Blood Mini Kit, Hilden, Germany). Three SNPs (rs179010, rs2074109, and rs179009) of the TLR7 gene were identified using PCR-based sequencing and the results were carried out in quality control as our previous study reported [29]. The amplicons were sequenced at the Sino Geno Max Co., Ltd, Beijing, China and SNPs were identified by the computer program of mutation surveyor V5.0.0 (SoftGenetics, USA).

### mRNA Expression of TLR7 in Peripheral Blood Mononuclear Cells (PBMCs)

The total RNA was extracted from PBMCs of healthy male individuals (N = 41, One of 121 samples was tested at three intervals) with TRIzol reagent (TransGen Biotech, Beijing, China). The relative expression of TLR7 mRNA was reverse transcribed with TransScript^®^ First-Strand cDNA Synthesis SuperMix (TransGen Biotech, Beijing, China) and obtained by using the 2-ΔΔCt method which was normalized with an endogenous control, β-actin. All assays were performed in triplicate.

### Serum Cytokine Profile of Healthy Male Individuals

Cytokines in the serum of healthy male individuals (N = 20, One of 58 samples was tested at three intervals) were analyzed with a multiplex immunoassay (Invitrogen, Vienna, Austria). The lower limits of detection (LOD) for the cytokines were: IFN-α, 0.2 pg/ml; IFN-γ, 0.2 pg/ml; IL-1α, 0.1 pg/ml; IL-1β, 0.2 pg/ml; TNF-α, 0.4 pg/ml; IL-6, 0.4 pg/ml; IL-10, 0.1 pg/ml; IL-12p 70, 0.04 pg/ml; IL-13, 0.1 pg/ml; IL-17A, 0.1 pg/ml; IL-4, 1.5 pg/ml; IL-8, 1.2 pg/ml; GM-CSF, 1.2 pg/ml; ICAM1, 76.3 pg/ml; IP-10, 0.3 pg/ml; MCP-1, 0.6 pg/ml; MIP-1α, 1.1 pg/ml; MIP-1β, 4.7 pg/ml; sE-Selectin, 555.3 pg/ml; sP-Selectin, 95.4 pg/ml.

### Statistical Analysis

Allele frequency was descriptively summarized, and each SNP was tested for deviation from Hardy-Weinberg equilibrium (HWE). Statistical analysis was carried out using SPSS statistical software version 17.0 (SPSS Inc., Chicago, USA). Differences in demographic data, allele frequencies, clinical parameters between different groups were evaluated by Student’s t-test, chi-square test, where appropriate. Logistic regression was used for the multivariate analyses, adjusted for age, and we then evaluated the independent contributions of the three SNPs to the susceptibility to and disease progression of HIV-1 infection with P values, odds ratios (ORs), and 95% confidence intervals (95% CIs). SHESIS online (http://analysis.bio-x.cn/myAnalysis.php) was used for the haplotype analysis. A two-sided P value of less than 0.05 was considered significant.

## Results

### Clinical Information of Study Subjects

**Figure 1:**
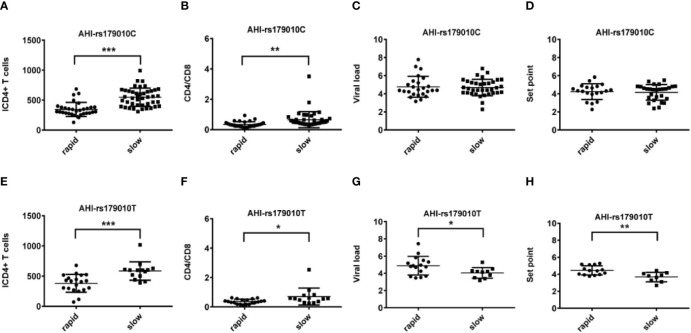
Relationship between clinical parameters and AHI patients with rapid/slow progression who carry rs179010C/T. **(A–H)** There are four clinical parameters including, ICD4 (initial CD4 T cells) **(A, E)**, CD4/CD8 (the ratio of CD4 versus CD8 T cells) **(B, F),** viral load **(C, G)**, and set point **(D, H)**. The y axis indicates the values of these four respective parameters. The x axis indicates two AHI patient groups with rapid or slow progression. The text on the top of each picture indicates allele rs179010C/T. Each dot represents an MSM individual, (●) represents individuals of AHI-rapid progress with TLR7 rs179010C/T allele. (■) represents individuals of AHI-slow progress with TLR7 rs179010C/T allele. * indicates P<0.05; **P < 0.01 and ***P < 0.001.

**Figure 2:**
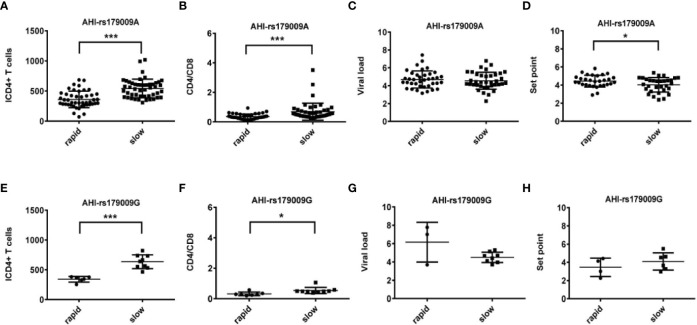
Relationship between clinical parameters and AHI patients with rapid/slow progression who carry rs179009A/G. **(A–H)** There are four clinical parameters including, ICD4 (initial CD4 T cells) **(A, E)**, CD4/CD8 (the ratio of CD4 versus CD8 T cells) **(B, F)**, viral load **(C, G)**, and set point **(D, H)**. The y axis indicates the values of these four respective parameters. The x axis, indicates two AHI patient groups with rapid or slow progression. The text on the top of each picture indicates allele rs179009A/G. Each dot represents an MSM individual, (●) represents individuals of AHI-rapid progress with TLR7 rs179009A/G allele. (■) represents individuals of AHI-slow progress with TLR7 rs179009A/G allele. *indicates P < 0.05; P < 0.01 and ***P < 0.001.

Demographic and clinical information of the patients and healthy controls are summarized in **Table 1**. There were significant differences in terms of age between patients and healthy controls (P = 0.000). No significant differences of age were observed between patients with AHI-rapid progression (AHI-rapid) and AHI-slow progression (AHI-slow) as well as between patients with AHI and CHI. We found significant differences in some clinical parameters between AHI patients with rapid and slow progression. For example, initial CD4 T-cell count (ICD4) and the ratio of CD4 versus CD8 T-cell counts (CD4/CD8) are significantly lower in the rapid progression group and higher in the slow progression group respectively (**Figures 1**, **2** and **5**).

**Table 1:** Demographic and clinical information of studied patients and healthy controls.

Characteristics	Acute HIV-1 infection	AHI- rapid progression	AHI- slow progression	Chronic HIV-1 infection	Cases	Healthy Controls
Patients (n)	157	54	60	113	270	196
Median age	32(20-56)	32(21-51)	33(20-53)	31(21-60)	31.5(20-60)	40(21-60)
(range, y)						
ICD4+ T cells	413(72-1323)	330(72-685)	558.5(302-1323)			
CD4+/CD8+	0.419(0.094-12.351)	0.309(0.094-0.944)	0.499(0.247-3.511)			
Viral load	4.696(2.283-7.777)	4.725(3.155-7.777)	4.406(2.283-6.776)			
(copies/ml in log)						
Set Point	4.438(2.267-5.834)	4.393(2.267-5.834)	4.164(2.382-5.502)			

ICD4+ T cells, initial CD4+ T cells.

### Relationship Between TLR7 Polymorphisms and Susceptibility to or Progression of HIV-1 Infection

The observed genotype distributions of these three SNPs were in agreement with the Hardy-Weinberg equilibrium (all of the P values >0.05). For TLR7 rs179010, the prevalence of minor allele T was significantly lower in patients than that of controls adjusted for age (32.6% vs 40.8%, P = 0.039) (**Table 2**). After subsets stratification, we found a much lower frequency of TLR7 rs179010 allele T in AHI patients (29.3% vs 40.8%, P = 0.002), especially in patients with AHI-slow progression (26.7% vs 40.8%, P = 0.015) than that of controls adjusted for age (**Tables 3** and **4**). The results indicated that TLR7 rs179010 was not only related to disease susceptibility but also disease progression. No significant differences in frequencies of TLR7 rs2074109 and rs179009 polymorphisms were observed when MSM patients or patients with AHI/AHI-slow/AHI-rapid were compared with controls (**Tables 2**–**4**). However, for TLR7 rs179009, the prevalence of minor allele G was significantly higher in CHI patients than that of controls adjusted for age (26.5% vs 14.3%, P = 0.016) (**Table 3**).

**Table 2:** The allele frequencies of three TLR7 SNPs in patients and controls.

Allele frequencies	Cases, N (%)	Controls, N (%)	OR (95% CI)	P value
	N = 270	N = 196		
TLR7 rs179010 major allele C	182(67.4)	116(59.2)	1	Ref.
TLR7 rs179010 minor allele T	88(32.6)	80(40.8)	0.798(0.631-0.988)	**0.039**
	N = 270	N = 196		
TLR7 rs2074109 major allele T	254(94.1)	186(94.9)	1	Ref.
TLR7 rs2074109 minor allele C	16(5.9)	10(5.1)	0.992(0.636-1.548)	0.973
	N = 270	N = 196		
TLR7 rs179009 major allele A	221(81.9)	168(85.7)	1	Ref.
TLR7 rs179009 minor allele G	49(18.1)	28(14.3)	1.037(0.776-1.386)	0.807

P value was adjusted for age by logistic regression between cases and controls. Bold value indicates the statistical significance.

**Table 3:** The allele frequencies of three TLR7 SNPs in patients with AHI/CHI and controls.

Allele frequencies	AHI Cases, N (%)	CHI Cases, N (%)	Controls, N (%)	P^1^ value/OR (95% CI)	P^2^ value/OR (95% CI)
rs179010	N = 157	No = 113	N = 196		
C	111(70.7)	71(62.8)	116(59.2)	1	1
T	46(29.3)	42(37.2)	80(40.8)	**0.002/**0.664 (0.513-0.860)	0.889/1.021 (0.765-1.362)
rs2074109	N = 157	No = 113	N = 196		
T	148(94.3)	106(93.8)	186(94.9)	1	1
C	9(5.7)	7(6.2)	10(5.1)	0.704/0.907(0.548-1.502)	0.612/1.154(0.664-2.004)
rs179009	N=157	No=113	N=196		
A	138(87.9)	83(73.5)	168(85.7)	1	1
G	19(12.1)	30(26.5)	28(14.3)	0.193/0.792(0.557-1.125)	**0.016/**1.526(1.081-2.153)

P^1/2^ value was adjusted for age by logistic regression between AHI/CHI cases and controls.

AHI, acute HIV-1 infection: CHI, chronic HIV-1 infection. Bold value indicates the statistical significance.

**Table 4:** The allele frequencies of three TLR7 SNPs in patients with AHI-rapid/slow and controls.

Allele frequencies	AHI-rapid, N (%)	AHI-slow, N (%)	Controls, N (%)	P^1^ value/OR (95% CI)	P^2^ value/OR (95% CI)
	N = 54	N = 60	N = 196		
TLR7 rs179010 C	34(63)	44(73.3)	116 (59.2)	1	1
TLR7 rs179010 T	20(37)	16(26.7)	80 (40.8)	0.276/0.822 (0.578–1.170)	**0.015/**0.638 (0.444–0.917)
	N=54	N=60	N = 196		
TLR7 rs2074109 T	51(94.4)	57(95)	186 (94.9)	1	1
TLR7 rs2074109 C	3(5.6)	3(5)	10 (5.1)	0.869/0.942 (0.461-1.924)	0.660/0.854(0.424-1.722)
	N=54	N=60	N=196		
TLR7 rs179009 A	47(87)	51(85)	168(85.7)	1	1
TLR7 rs179009 G	7(13)	9(15)	28(14.3)	0.799/0.937(0.574-1.532)	0.614/0.891(0.568-1.396)

P^1/2^ value was adjusted for age by logistic regression between AHI-rapid/slow cases and controls.

AHI-rapid, AHI patients with rapid progression: AHI-slow, AHI patients with slow progression. Bold value indicates the statistical significance.

Subsequently, we performed haplotype analysis with rs179010C/T, rs2074109T/C and rs179009A/G. All those of frequency<0.03 were ignored in analysis. Four major haplotypes CTA, TTA, CTG, and CCA were found and listed in **Table 5**. We found a significant difference in haplotype TTA frequency of cases compared with that of controls (32.6% vs 40.3%, P = 0.013). Especially AHI patients had a much lower frequency of TTA haplotype(29.3%, P = 0.002). In contrast, a higher frequency of haplotype CTA was found in AHI patients than in controls (52.9% vs 40.3%, P = 0.001). Further, the possible influence of TLR7 rs179010 was confirmed by haplotype CTA and TTA distribution in AHI-slow patients and controls (P = 0.008 for haplotype CTA; P = 0.002 for haplotype TTA), but not in AHI-rapid patients (**Table 5**). The data present here suggested that the haplotype TTA might be associated with decreased disease susceptibility and even related to slow progression. Besides, the possible influence of TLR7 rs179009 was confirmed by haplotype CTA and CTG distribution in CHI patients and controls (P = 0.000 for haplotype CTA; P = 0.000 for haplotype CTG) (**Table 5**).

**Table 5:** Haplotype analysis of TLR7 rs179010(C/T), rs2074109(T/C) and rs179009(A/G) in controls and cases/CHI/AHI/AHI-rapid/AHI-slow patients.

Haplotype	AHI (%)	AHI-rapid (%)	AHI-slow (%)	CHI (%)	Cases (%)	Controls (%)	P^a^	P^b^	P^c^	P^d^	P^e^
CTA	52.9	43.4	54.4	30.1	43.3	40.3	**0.001**	0.593	**0.008**	**0.000**	0.390
TTA	29.3	37.7	24.6	37.2	32.6	40.3	**0.002**	0.605	**0.002**	0.054	**0.013**
CTG	12.1	13.2	15.8	26.5	18.1	13.8	0.495	0.865	0.602	**0.000**	0.080
CCA	5.7	5.7	5.3	6.2	5.9	5.1	0.724	0.827	0.954	0.822	0.601

a P value calculated between AHI patients and controls.

b P value calculated between AHI-rapid patients and controls.

c P value calculated between AHI-slow patients and controls.

d P value calculated between CHI patients and controls.

e P value calculated between cases and controls. Bold value indicates the statistical significance.

### TLR7 rs179010T With Low Viral Load in AHI-Slow Patients and TLR7 rs179009A With High Set Point in AHI-Rapid Patients

We found significant differences between AHI patients with rapid and slow progression with initial CD4 T-cell count (ICD4) and the ratio of CD4 versus CD8 T-cell count (CD4/CD8). The differences of these parameters are independent of TLR7 alleles (**Figures 1** and **2**). However, other parameters show a specific relation to certain alleles.

**Figure 3:**
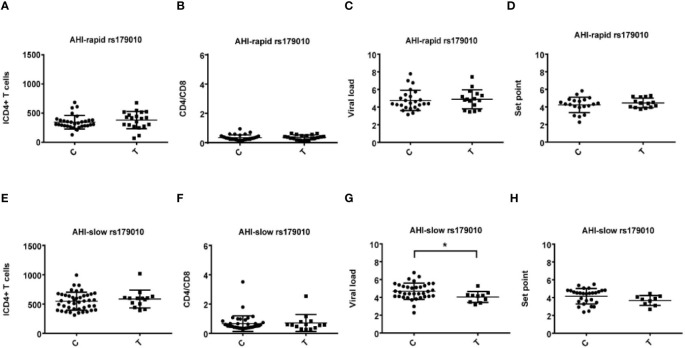
Relationship between rs179010C/T and clinical parameters in AHI patients with rapid/slow progress. **(A–H)** There are four clinical parameters including, ICD4 (initial CD4 T cells) **(A, E)**, CD4/CD8 (the ratio of CD4 versus CD8 T cells) **(B, F)**, viral load **(C, G)**, and set point **(D, H)**. The y axis indicates the values of these four respective parameters. The x axis, indicates two patient groups with TLR7 rs179010C/T allele. The text on the top of each picture represents AHI-rapid/slow patients. Each dot represents an MSM individual, (●) represents individuals of AHI-rapid/slow progress with TLR7 rs179010C allele. (■) represents individuals of AHI-rapid/slow progress with TLR7 rs179010T allele. *indicates P < 0.05; P < 0.01 and P < 0.001.

We found correlations of the viral load (P = 0.035) and set point (P = 0.003) with rs179010T based on the disease progression. However, no similar correlation was found in rs179010C carriers (**Figure 1**). It showed that the TLR7 rs179010T allele was significantly associated with a lower viral load in AHI-slow patients (P = 0.042) (**Figure 3**). A similar trend was in the analysis of the set point, but the difference did not reach significance (**Figure 3**). The results imply a functional influence of rs179010 on TLR7 in AHI patients, especially in AHI-slow patients. The data suggest a strong effect of TLR7 rs179010 in the initial stages of an HIV-1 infection.

**Figure 4:**
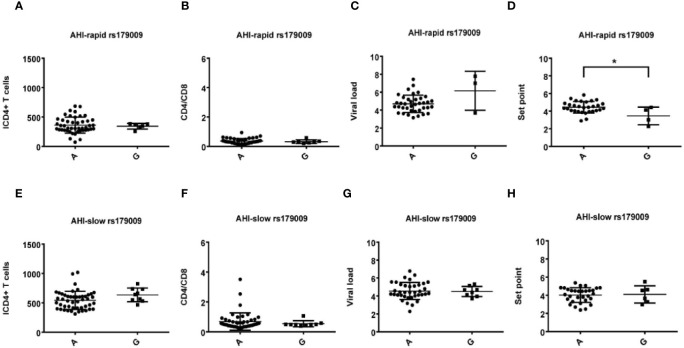
Relationship between rs179009A/G and clinical parameters in AHI patients with rapid/slow progress. **(A–H)** There are four clinical parameters including, ICD4 (initial CD4 T cells) **(A, E)**, CD4/CD8 (the ratio of CD4 versus CD8 T cells) **(B, F)**, viral load **(C, G)** and set point **(D, H)**. The y axis indicates the values of these four respective parameters. The x axis, indicates two patient groups with TLR7 rs179009A/G allele. The text on the top of each picture represents AHI-rapid/slow patients. Each dot represents an MSM individual, (●) represents individuals of AHI-rapid/slow progress with TLR7 rs179009A allele. (■) represents individuals of AHI- rapid/slow progress with TLR7 rs179009G allele. * indicates P < 0.05; P < 0.01 and P < 0.001.

**Figure 5:**
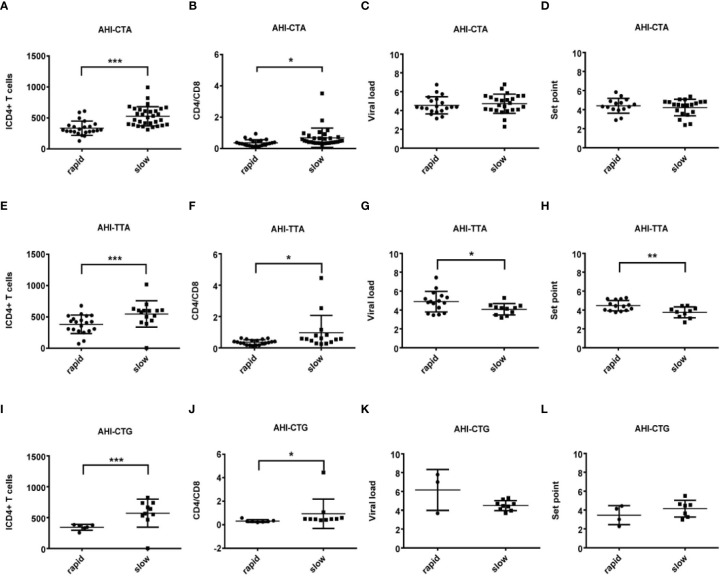
Relationship between clinical parameters and AHI patients with rapid/slow progression who carry different haplotypes (CTA/TTA/CTG). **(A–H)** There are four clinical parameters including, ICD4 (initial CD4 T cells) **(A, E, I)**, CD4/CD8 (the ratio of CD4 versus CD8 T cells) **(B, F, J)**, viral load **(C, G, K)** and set point **(D, H, L)**. The y axis indicates the values of these four respective parameters. The x axis, indicates two patient groups of AHI with rapid and slow progression. The text on the top of each picture represents haplotype CTA/TTA/CTG. Each dot represents an MSM individual, (●) represents individuals of AHI-rapid progress with TLR7 CTA/TTA/CTG haplotype. (■) represents individuals of AHI-slow progress with TLR7 CTA/TTA/CTG haplotype. * indicates P < 0.05; **P < 0.01 and ***P < 0.001.

**Figure 6:**
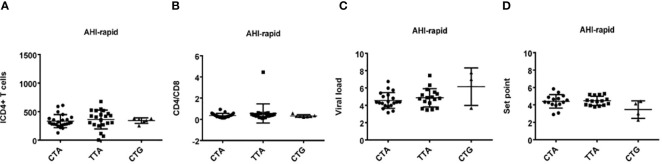
Relationship between haplotypes (CTA/TTA/CTG) and clinical indexes in AHI patients with rapid progression. **(A–D)** There are four clinical parameters including, ICD4 (initial CD4 T cells) **(A)**, CD4/CD8 (the ratio of CD4 versus CD8 T cells) **(B)**, viral load **(C)** and set point **(D),** The y axis indicates the values of these four respective parameters. The x axis, indicates three AHI–rapid patient groups with TLR7 CTA/TTA/CTG haplotype. Each dot represents an MSM individual, (●) represents individuals with TLR7 CTA haplotype. (■) represents individuals with TLR7 TTA haplotype. (▲) represents individuals with TLR7 CTG haplotype.

**Figure 7:**
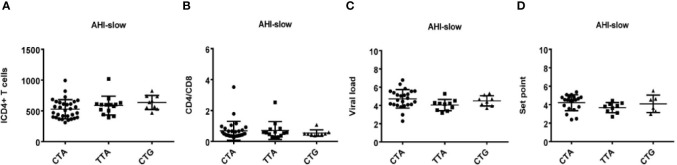
Relationship between haplotypes (CTA/TTA/CTG) and clinical indexes in AHI patients with slow progression. **(A–D)** There are four clinical parameters including, ICD4 (initial CD4 T cells) **(A)**, CD4/CD8 (the ratio of CD4 versus CD8 T cells) **(B)**, viral load **(C)** and set point **(D)**. The y axis represents the values of these four clinical parameters respectively. In x axis, there are three AHI–slow patient groups with TLR7 CTA/TTA/CTG haplotype. Each dot represents an MSM individual, (●) represents individuals with TLR7 CTA haplotype. (■) represents individuals with TLR7 TTA haplotype. (▲) represents individuals with TLR7 CTG haplotype.

Otherwise, we also found that TLR7 rs179009A was significantly related to a high set point in AHI-rapid patients (**Figures 2** and **4**), indicating that carriers with wild type TLR7 rs179009A are prone to rapid progress and poor prognosis. The correlation analysis between clinical parameters and TLR7 haplotypes (CTA/TTA/CTG) still support the conclusion of single genotypic analysis (**Figures 5**–**7**).

### mRNA Expression of TLR7 and Cytokine Profile in Healthy Male Individuals

We directly analyzed the mRNA expressions of TLR7 in peripheral blood mononuclear cells of 41 healthy male individuals, that included 26 rs179010C and 15 rs179010T carriers, 38 rs2074109T and 3 rs2074109C carriers as well as 31 rs179009A and 10 rs179009G ones. No significant difference was found in TLR7 mRNA expressions between these three kinds of SNP carriers (P>0.05) in healthy male individuals (**Supplementary Figure 1**).

We also detected 20 cytokines (CKs) in serum of healthy male subjects (N = 20) with 13 major allele rs179010C and 7 minor rs179010T, as well as 15 major allele rs179009A and 5 minor rs179009G. The CKs level of subjects carrying the major alleles were still not significantly different with those of subjects carrying the minor alleles (P>0.05) (**Supplementary Figures 2** and **3**).

Together, there was no significant difference found either in the TLR7 mRNA level or serum cytokine proﬁle of healthy male individuals with different alleles analyzed.

## Discussion

In this study, we found that genetic variation of TLR7 plays an important role in HIV-1 infection. To our knowledge, this is the first report to show the relationship between TLR7 polymorphisms and susceptibility to and disease progression of an HIV-1 infection in Chinese people, especially in Chinese MSM population with AHI and CHI. Similar studies on the association of these two polymorphisms (rs179010 and rs179009) with HIV infection have not been reported in other ethnic populations.

It was considered that HIV-1 goes under the radar of innate immune detection and hence evades Interferon (IFN) restriction in the eclipse phase that follows virus inoculation, allowing it to establish infection in host cells. Innate immune responses that link induction of IFN and activation of the inflammasome may contribute to the host control of viremia. Activation of the TLR7 signaling pathway can facilitate the production of antiviral cytokines, including IFNs, TNF-α, interleukin-6 (IL-6) and IL-12, which are critical in the development of efficient antiviral immune responses, including the production of antiviral antibodies (31, 32). These early cytokines and the secretory cells, such as dendritic cells (DCs), are pivotal in shaping the immune responses that develop in acute or early HIV-1 infection (33). Endocytosis of HIV-1 activates pDCs *via* interactions of TLR7 and viral RNA (17, 18), and TLR7 persistent activation also contributes to chronic activation of the immune system which is closely related with AIDS progress (34–36). TLR7 shows the characteristics of a double-edged sword, especially in different clinical phases after HIV infection. GS-9620, an oral agonist of TLR7, activated HIV production (37). However, it suppressed the hepatitis B virus (HBV) in chimpanzees of a chronic infection (38). In acute HIV infection, the antiviral state induced by TLR7/8 may restrict overwhelming HIV replication. In contrast, triggering TLR7/8 induces HIV virions to release in latently infected cells.

In this study, we assessed the impact of three intronic SNPs of TLR7 on susceptibility to and disease progression of an HIV-1 infection in Chinese MSM. Based on SNP function prediction (http://snpinfo.niehs.nih.gov), mutations of TLR7 rs179009 and 179010 are predicted to occur at transcription factor binding sites (TFBSs), which could affect the level and/or timing of TLR7 expression and result in a difference in the production of downstream cytokines such as IFN-α. Another SNP rs2074109, which is not predicted as a TFBS, is located in 31 nucleotide upstream of rs179009. No relation was found in TLR7 rs2074109 with disease susceptibility and progression of chronic HBV infection in Chinese patients in our previous study (29).

We found that TLR7 rs179010 variation seems to be associated with acute stage of HIV-1 infection, while the variation of rs179009 was closely related to the chronic stage of HIV-1 infection. In the present study, Chinese MSM with AHI, but not those with CHI, had a significantly lower frequency in minor allele T of TLR7 rs179010 compared to that of healthy controls (P = 0.002). Further, a much lower frequency of this allele was present in patients with AHI-slow, indicating a potential effect of TLR7 rs179010 minor allele T against susceptibility and progression of the acute HIV-1 infection. Further, the allele was also found to be associated with a lower viral load in patients with AHI-slow. These results imply that TLR7 rs179010T might reduce the risk of disease by participating in inhibition of viral load, or even in the case that an infection has already established thus leading to slower progression in the early phase of HIV-1 infection.

Association of the TLR7 rs179010 minor allele T has been reported with several other viral diseases, for example chronic HBV infection in Chinese males (29), chikungunya virus infection in Indian males (39) and dengue virus infection in Indian patients (40). Recently, this allele of TLR7 rs179010 has been also found to be a risk factor for EV-A71–related HFMD in Chinese male children (41).

TLR7 can recognize HCV RNAs, leading to the production of IFN and antiviral cytokines to influence HCV infection and progression (42). TLR7 rs179009, but not rs179010, influenced chronic HCV infection with gender difference (27, 28, 43). Yue et al. reported that Chinese female patients who carry TLR7 rs179009 GG genotype and haplotype GCG of rs179009, rs179010 and rs179012 had an increased susceptibility to HCV infection (27). In Moroccan subjects, carriage of the TLR7 rs179009 major allele A was associated with a two-fold increase in spontaneous HCV clearance in female patients, but not in male patients. And TLR7 rs179009 minor allele G increased the risk of disease progression in both sexes (44). We previously reported that TLR7 rs179009 minor allele G plays an important role in the progression of chronic HBV infection to the related liver cirrhosis and hepatocellular carcinoma in Chinese male adults (29). In this study, we observed that the minor allele G was over-present in Chinese MSM with CHI, but not in those with AHI. Although the pathogenesis of HCV, HBV and HIV infections was quite different, the role played by genetic variation of TLR7 rs179009 seemed to be similar in the courses of certain chronic diseases, which might be due to the effect of TLR7 on host immune response, especially during the chronic inflammatory process. Interestingly, we also found that TLR7 rs179009 major allele A, as a potential risk factor, might relate to the prognosis of AHI-rapid male patients through adjusting the set point.

No significant difference was found in the mRNA level of TLR7 and the related cytokines secretion from directly testing PBMCs and serum of healthy males who carried different alleles (rs179010C/T and rs179009A/G) in this study, indicating these two polymorphisms do not affect the normal expression of TLR7 and normal cytokine profile of healthy people. It is consistent with previous tests on TLR7 rs179010 mRNA in healthy individuals, and also on the secretion of IFN- α and IL-6 among different rs179010 allele carriers after ex vivo stimulation with TLR7 ligand (27). However, Wang et al. found that variation of TLR7 rs179009 might impair immune responses during HCV infection in male Taiwanese (28). Cells from whole blood of male healthy subjects with TLR7 rs179009 major allele A produced more IFN-α and fewer amounts of IL-1β upon stimulation, while cells with TLR7 rs179009 minor allele G produced significantly low IFN-α and high amounts of IL-1β. Another report showed that the rs179009 minor allele G variant was associated with persistent HCV infection in Chinese females and led to a decreased production of IFN-α after ex vivo stimulation (27).

Except for some polymorphisms of TLR2, 3, 4, 8, and 9 (19–24), there have been only two reports on HIV infection with exonic TLR7 Gln11Leu, which shows a relation to the diminution of IFNα induction, but this polymorphism rs179008 is absent in Chinese Han people (45). Based on the data of ALFA Allele Frequency (New)/Hapmap/1000Genomes from dbSNP, allele frequency of rs179010/rs179009 in European, African, Asian/East Asian people are apparently different. The frequency of TLR7 rs179010 allele T in European, African, Asian/East Asian people are 31%-32.4%, 11%-15.8% and 31.7%-43.2% respectively, while the frequency of TLR7 rs179009 allele G in European, African, Asian/East Asian people are 19.8%-22.2%, 12.7%-16.5% and 5%-15.7% respectively. Djin-Ye Oh suggested that IFNα production is of importance for the control of HIV replication during early, rather than in the late disease stage (25). In the present study, we found an association of TLR7 rs179010 minor allele T with low viral load in the acute phase of HIV-1 infection, which might be related to IFNα production. Therefore, we speculate that this TFBS polymorphism affects the timing of TLR7 expression, which need stimulation to elicit TLR7 expression and the following high secretion of IFNα in acute phase of HIV-1 infection. We also found that the TLR7 rs179009 major allele A seems to function as a risk factor for rapid progress and poor prognosis in the acute phase of HIV-1 infection, while its minor allele G could be a risk factor for the chronic course of HIV-1 infection. The previous study suggested that TLR7 rs179009G might impair the function of TLR7 with reduction of IFNα production and with high-level secretion of downstream inflammatory cytokines. Those may influence the risk of persistent virus infection and disease progression with persistent immune activation, including HCV, HBV, and HIV-1. Although the detailed mechanism of these TLR7 SNPs on different viral diseases is not clear, combined with SNP functions found in previous studies, these two TLR7 intronic SNPs may play critical roles in various viral diseases (44, 46) and play different roles in acute/chronic disease phases. Further functional experiments need to verify our hypothesis. Both of PBMCs/whole blood from patients with different clinical stages should be tested in ex vivo stimulation experiments based on disease characteristics.

There are certain limitations to this study. The first was a relatively small number of subjects included in each group, especially the number of patients with AHI-slow/rapid progression. Second, only three intronic SNPs of TLR7 were assessed. Although we found the difference in frequency of haplotype TTA between patients with AHI and CHI, we could not exclude the possibility that other intronic SNPs in TLR7, which are not included in this present study could contribute to the difference observed. Third, we did not perform *ex vivo* stimulation tests of blood samples collected from patients to show effects of the three intronic SNPs on functionality of TLR7 such as mRNA expression and related cytokine profiles.

In summary, our study shows strong correlation of TLR7 rs179010 minor allele T and haplotype TTA with decreased susceptibility and slow progression of HIV-1 infection in acute phase, which could be partly due to restriction of viral load, and influence of the set point. While TLR7 rs179009 minor allele G and haplotype CTG over-presented in patients of CHI, inferred as “a risk factor” in the chronic stage of an HIV infection. This finding supports a role for genetic variations of TLR7 in susceptibility and disease progression of an HIV-1 infection and warrants further studies on the effect of TLR7 polymorphisms on HIV-1 infection in different populations.

## Data Availability Statement

The raw data supporting the conclusions of this article will be made available by the authors, without undue reservation.

## Ethics Statement

The studies involving human participants were reviewed and approved by the Beijing Youan Hospital Research Ethics Committee. The patients/participants provided their written informed consent to participate in this study.

## Author Contributions

QH, HW, JZ, and TZ conceived, designed and supervised the study. JZ, BS, LC, ZL, HHW, XH, KZ, AL, NC, LL, and WX collected samples and performed the experiments. JZ, TZ, HW, and QH analyzed the data and wrote the paper. All authors contributed to the article and approved the submitted version.

## Funding

This work was partly supported by the National 13th Five-Year Grand Program on Key Infectious Disease Control (2017ZX10202101-004-001 to TZ, 2017ZX10202102-005-003 to BS), Beijing Natural Science Foundation (7172016 to JZ), the NSFC-NIH Biomedical collaborative research program (81761128001 to HW), the Beijing Municipal of Science and Technology Major Project (D161100000416003 to HW), and the Beijing Key Laboratory for HIV/AIDS Research (BZ0089).

## Conflict of Interest

The authors declare that the research was conducted in the absence of any commercial or financial relationships that could be construed as a potential conflict of interest.
