# Supplementary material for: Effects of TLR7 Polymorphisms on the Susceptibility and Progression of HIV-1 Infection in Chinese MSM Population
Source: Front Immunol. 2020 Oct 26;11:589010. doi: 10.3389/fimmu.2020.589010 (PMC7649213; doi:10.3389/fimmu.2020.589010)
Supplement: Supplementary file 1 [file DataSheet_1.docx]

Supplementary Material

**Supplementary Figure 1.** The figure legends are required to have the same font as the main text, 12 point normal Times New Roman, single spaced. Please use a single paragraph for each legend and prepare the figures keeping in mind the PDF layout.

**(A) (B) (C)**

**Supplementary Figure 1 TLR7 mRNA expression of healthy male individuals who carry rs179010C/T, rs2074109T/C, rs179009A/G by quantitative RT-PCR.**

The total RNA was extracted from PBMCs of healthy male volunteers. The relative expression of TLR7 mRNA was reverse transcribed and obtained by using the 2^-∆∆Ct^ method which was normalized with an endogenous control, β-actin. Each dot represents an individual, (●) represent individuals with TLR7 rs179010C/rs2074109T/rs179009A major allele, (■) represent individuals with TLR7 rs179010T/rs2074109C/rs179009G minor allele. N=41. The value of each dot represents the mean of three independent tests and the horizontal bars represent mean value of this group.

**(A) (B) (C) (D)**

**(E) (F) (G) (H)**

**(I) (J) (K) (L)**

**(M) (N) (O) (P)**

**(Q) (R) (S) (T)**

**Supplementary Figure 2 cytokine profile in serum of healthy male individuals who carry rs179010C/T by a multiplex immunoassay.**

Each dot represents an individual, (●) represent individuals with TLR7 rs179010C major allele, (■) represent individuals with TLR7 rs179010T minor allele. N=20. The horizontal bars represent mean value.

**(A) (B) (C) (D)**

**(E) (F) (G) (H)**

**(I) (J) (K) (L)**

**(M) (N) (O) (P)**

**(Q) (R) (S) (T)**

**Supplementary Figure 3 cytokine profile in serum of healthy male individuals who carry TLR7 rs179009A/G by a multiplex immunoassay.**

Each dot represents an individual, (●) represent individuals with TLR7 rs179009A major allele, (■) represent individuals with TLR7 rs1790009G minor allele. N=20. The horizontal bars represent mean value.
